# Extracellular Vesicles in Acute Stroke Diagnostics

**DOI:** 10.3390/biomedicines8080248

**Published:** 2020-07-28

**Authors:** Katrine Tang Stenz, Jesper Just, Rolf Ankerlund Blauenfeldt, Kim Ryun Drasbek

**Affiliations:** 1 Center of Functionally Integrative Neuroscience, Department of Clinical Medicine, Aarhus University, DK-8000 Aarhus, Denmark; kts@cfin.au.dk (K.T.S.); jesperj@cfin.au.dk (J.J.); 2 Sino-Danish Center for Education and Research, Beijing 101400, China; 3 Sino-Danish Center for Education and Research, DK-8000 Aarhus, Denmark; 4 Department of Neurology, Aarhus University Hospital, DK-8200 Aarhus, Denmark; rolfblau@rm.dk; 5 Department of Clinical Medicine, Aarhus University, DK-8200 Aarhus, Denmark

**Keywords:** stroke, circulating biomarkers, extracellular vesicles, diagnostics

## Abstract

There is a large unmet need for fast and reliable diagnostics in several diseases. One such disease is stroke, where the efficacy of modern reperfusion therapies is highly time-dependent. Diagnosis of stroke and treatment initiation should be performed as soon as possible, and preferably before arrival at the stroke center. In recent years, several potential blood biomarkers for stroke have been evaluated, but without success. In this review, we will go into detail on the possibility of utilizing extracellular vesicles (EVs) released into the blood as novel biomarkers for stroke diagnostics. EVs are known to reflect the immediate state of the secreting cells and to be able to cross the blood–brain barrier, thus making them attractive as diagnostic biomarkers of brain diseases. Indeed, several studies have reported EV markers that enable differentiation between stroke patients and controls and, to a lesser extent, the ability to correctly classify the different stroke types. Most of the studies rely on the use of sophisticated and time-consuming methods to quantify specific subpopulations of the nanosized EVs. As these methods cannot be easily implemented in a rapid point of care (POC) test, technical developments followed by prospective clinical studies are needed.

## 1. Introduction

Stroke is the second-leading cause of death worldwide and a leading cause of long-term disability [1]. The most common type is acute ischemic stroke (AIS), which occurs in 85% of cases, with the remaining cases being hemorrhagic strokes dominated by spontaneous intracerebral hemorrhage (ICH) [2]. Early, fast and precise diagnosis is paramount for optimal treatment selection in acute stroke patients to preserve salvageable ischemic brain tissue in AIS, and to prevent hematoma expansion in ICH and ultimately improve functional outcome [2]. Reducing time to treatment is vital. In large vessel occlusion (LVO) stroke, each minute saved between symptom onset and treatment initiation is estimated to save 1.9 million neurons and grant 4.2 days of extra healthy life [3,4]. Clinical examination alone cannot reliably differentiate between AIS and ICH, making neuroimaging mandatory before treatment initiation [2]. Currently, no effective treatment can be initiated in the hyper-acute prehospital phase after stroke onset [5]. Thus, stroke center admission, performing and interpreting neuroimaging results are needed before treatment initiation. There is an urgent need for fast and reliable acute stroke diagnostics to fully harness the effect of current reperfusion therapies and to allow future neuroprotective strategies to be started as soon as possible [6]. For this purpose, we hypothesize that blood-derived extracellular vesicles (EVs) can be used to discriminate between stroke types as well as give an indication of the current cerebrovascular disease state.

## 2. Stroke Pathophysiology and Current Treatment

The two major forms of stroke are AIS, a thrombotic or thromboembolic blockage of a blood vessel, and ICH, typically caused by the spontaneous rupture of a small cerebral blood vessel [5]. In transient ischemic attack (TIA), blood flow is temporarily interrupted and restored before causing lasting (>24 h) neurological deficits and brain tissue injury.

In ischemic stroke, the downstream lack of blood flow, and thereby oxygen and nutrients, leads to a cascade of responses, cumulating in neuronal cell death [5]. Most ischemic strokes are caused by a thromboembolic event originating from a ruptured plague in large artery atherosclerotic disease or as a cardioembolic stroke caused by dislodged embolisms formed in the heart. Another frequent stroke etiology is cerebral small vessel disease (cSVD), where thrombotic occlusion of the small penetrating cerebral arteries leads to a lacunar stroke [5]. In AIS, the occlusion of a vessel results in a downstream area of critically hypoperfused brain tissue and a surrounding area of impaired, yet salvageable, tissue known as the “ischemic penumbra” [7]. The preeminent aim is to achieve complete reperfusion of the occluded vessel as soon as possible to save the ischemic penumbra [8], [9]. Reperfusion therapies, that pharmacologically dissolve the blood clot (tissue type plasminogen activators), or endovascular intervention (mechanical thrombectomy), have greatly improved the outcome after AIS [10,11,12]. However, the treatment effect rapidly declines from the first hour after symptom onset and must be started within 4.5–6 h (in some cases up to 24 h) to prevent the evolution of the infarct core [12,13,14,15]. Currently, only a minority of patients are treated with reperfusion therapies, mainly due to too-late arrival at the stroke center, exceeding the optimal treatment windows [16,17].

Acute blood pressure lowering has been the mainstay of ICH management since 2013, while this treatment may be harmful in AIS patients [18,19,20]. Clinical examination alone cannot reliably differentiate between AIS and ICH, highlighting the need for early stroke diagnostics [2].

## 3. Current Stroke Diagnostics

The clinical presentation of stroke involves the sudden onset of a focal neurological deficit from the central nervous system (CNS). Symptoms are numerous and can include hemiparesis, facial palsy, hemisensory disturbances and/or speech difficulties [5].

The diagnosis of stroke requires the ability to (1) differentiate stroke from stroke mimics (migraine, seizures with vestibular disturbances etc.) and (2) discriminate ischemic from hemorrhagic stroke. Currently, the latter is only possible after neuroimaging (Computer Tomography (CT) or Magnetic Resonance Imaging (MRI)) has been obtained and interpreted at the stroke center [2]. To reduce time to treatment initiation, mobile stroke units (MSUs) equipped with an on-board (CT) scanner that enables prehospital diagnosis of ischemic and hemorrhagic stroke, and thereby treatment initiation in the ambulance, have been developed. This ultra-early treatment initiation has been associated with improved functional outcome compared to in-hospital thrombolysis [21,22]. However, MSUs may only be cost-efficient in large metropolitan areas. As an alternative, point of care (POC) devices using either transcranial Doppler ultrasonography, electric impedance or microwave tomography have been designed to diagnose ischemic and hemorrhagic stroke in the field, but none have reached clinical practice [6,23].

Several clinical (prehospital) stroke scores have already been implemented with the aim of better identifying patients with a putative stroke, and in particular patients with LVO stroke, who are eligible to direct transfer to a mechanical thrombectomy capable stroke center [24,25]. The diagnostic performance and complexity vary between scores, and only a few have been prospectively validated in the prehospital field [24,25]. In-hospital stroke severity is quantified using the more detailed National Institute of Health Stroke Scale (NIHSS) score, with scores ranging from 0 to 42, and NIHSS scores ≥ 6–10 identifying patients with a moderate to severe stroke with increased likelihood of having an underlying LVO [2,26]. Furthermore, diagnostic performance is far from optimal, and it is not possible to differentiate ischemic from hemorrhagic stroke based on a clinical score alone.

Thus, there is a great need for readily available and reliable non-clinical biomarkers as aids in stroke diagnosis [27].

## 4. Circulating Brain Biomarkers

The highly selective, semi-permeable blood–brain barrier (BBB) controls the exchange of substances between the brain and blood, and, in this way, protects the brain against the invasion of pathogens and pathological compounds from the blood. On the other hand, the tight nature of the BBB also hinders the release of CNS-specific biomarkers into the blood, making circulating CNS markers scarce and therefore difficult to detect. The BBB is composed of endothelial cells, pericytes, astrocytes, neurons, and the extracellular matrix (ECM), collectively known as the neurovascular unit (NVU) [28]. The integrity of the BBB is mainly supported by the tight junctions between endothelial cells [29]. During an ischemic stroke the oxygen and nutrient depletion leads to energy failure and the swelling of endothelial cells, which causes them to lose contact with each other and to astrocytic endfeet [30]. In combination with ECM degradation, this leads to apoptotic cell death in the NVU, disintegration of the BBB and, ultimately, evolution of the ischemic core [5]. For hemorrhagic stroke, the BBB is compromised as soon as the hematoma emerges. In other words, the cascade which follows BBB disruption is a gradual degradation process and, consequently, the release of different biomarkers might change over time.

Following the initial disruption of the BBB, toxic free radicals, proteins, lipids, and microRNA are released in the blood [5,30]. Utilizing these as circulating biomarkers could provide a dynamic and powerful tool for disease screening, diagnosis, prognosis, and treatment efficacy monitoring. To date, many targets have been investigated including inflammatory markers, cytokines, growth factor-like molecules, hormones, lipids and microRNAs [31], but unfortunately, none of these have reached clinical practice [6]. Most of these studies focused on the diagnostic and/or prognostic abilities of a single biomarker in blood samples drawn after the hyper-acute phase (prehospital), and without being combined with a clinical stroke severity assessment in the prehospital phase or acute neuroimaging.

In order to use biomarkers as acute diagnostic tools, it is paramount that they are (1) released in the hyper-acute phase and continue to be so consistently over time; (2) unique in terms of composition both from other stroke subtypes and stroke mimics; and (3) can be easily and consistently measured with relatively simple devices.

Thus, circulating biomarkers to be used in the prehospital phase as POC measurements are a large unmet need in acute stroke care. EVs are interesting candidates as bearers of circulating biomarkers, due to their high stability in the blood, ability to cross an intact BBB and their uniqueness in terms of surface proteins and cargo, making them highly relevant biomarkers for stroke diagnostics.

## 5. Extracellular Vesicles

EVs are a diverse group of cell-derived, membrane-enclosed vesicles, which cannot independently replicate [32]. They are characterized according to their physical characteristics, biochemical composition and origin. They can be subdivided into multiple categories: two of these are exosomes and ectosomes (also named microvesicles/microparticles (MPs)). Exosomes are the smallest category of EVs; they range from 30–100 nm in diameter [33] and are released from multivesicular bodies (MVB) when these fuse with the plasma membrane of the donor cell [34]. Ectosomes, which are larger irregular-shaped vesicles, ranging from 50–1000 nm, are released by the outward budding of a small part of the plasma membrane upon cell activation or injury [35]. In this review, EVs will be used as a common label of all secreted vesicles, as is recommended by the International Society for Extracellular Vesicles (ISEV), when the origin of the EVs is ambiguous [32].

Common for all EVs are the secretion into extracellular fluids and biofluids, e.g., blood and cerebrospinal fluid (CSF) [36]. Upon secretion, EVs are involved in waste-removal and cell-to-cell communication, even over long distances, and through the BBB and the blood–cerebrospinal-fluid barrier [37,38,39]. EVs display specific surface markers, which have been proposed to be responsible for their intrinsic homing [40]. When EVs reach the target cell they are taken up by various endocytotic pathways, including clathrin-dependent endocytosis, and clathrin-independent pathways such as macropinocytosis and phagocytosis, among others [41]. After uptake, the EVs release their cargo composed of lipids, proteins, and nucleic acids [42].

### Extracellular Vesicles in CNS Pathology

The cargo, unique surface markers and release of EVs are altered in accordance with their cellular origin and the physiological/pathological state of the secreting cell [43]. Furthermore, EVs can cross the intact BBB, which may be a key feature for optimal stroke biomarkers. The disruption of BBB integrity occurs late in the treatment window of AIS and is associated with reduced treatment efficacy and higher rates of hemorrhagic complications [44,45]. The first stages of BBB breakdown during an ischemic stroke is associated with reduced oxygen and nutrient availability, which leads to ATP energy disruption and the disruption of homeostasis due to intracellular cation accumulation (i.e., Na^+^) [46]. After this, endothelial cells will start to swell and lose contact at tight junctions, which will lead to the BBB becoming leaky. As BBB breakdown is preceded by distinct physiological processes, EVs released from the initial affected cells, e.g., endothelial cells may provide an early and unique secretion profile. Thus, EVs may provide a unique molecular window to the brain, while the BBB is still intact and tissue is still salvageable. It opens the possibility of using EV biomarker panels to distinguish between different CNS states as well as acute diseases [47]. Recently, the role of EVs in stroke pathogenesis, diagnosis and as future treatment candidates has been a topic of increased interest [48,49]. However, to date, no studies have examined the expression profiles of circulating EVs in the hyper-acute prehospital phase in patients with a putative stroke. Such EV profiles could be used to discriminate between stroke types (ischemic vs. hemorrhagic) as well as give a clear indication of the current cerebrovascular disease state and aid in determining stroke etiology. By utilizing the inherent benefits of EVs as circulating CNS biomarkers, stroke diagnosis might be feasible even before hospital admission.

## 6. EV Isolation and Characterization

EV isolation and a subsequent investigation of the physicochemical properties, such as concentration, size and surface charge, play a central role in precise determination of EV characteristics and their diagnostic value [50]. For EV isolation from plasma samples, classical methods include differential centrifugation (ultracentrifugation and density-gradient ultracentrifugation), ultrafiltration, size exclusion chromatography, immunocapture, and polymer-based precipitation [51]. Each of these methods have distinct advantages and disadvantages, which have been described and compared in detail elsewhere [52]. For the characterization and validation of the isolated EVs, a series of techniques is regularly applied. EV concentration and size distribution can be measured by nanoparticle tracking analysis (NTA), dynamic light scattering (DLS), or tunable-resistive pulse sensing (TRPS), and then be cross-validated by electron microscopy or atomic force microscopy. Furthermore, EV identity is often validated by the presence of the classical tetraspanin EV markers CD9, CD63, and CD81. The establishment of canonical EV markers, however, has proven difficult, pointing to a pronounced heterogeneity of secreted EVs [53]. Thus, it is necessary to thoroughly characterize EVs obtained under specific experimental settings.

Advances within flow cytometry have increased the importance of this method within EV research. With an increase in sensitivity, vesicles down to 100 nm in diameter can now be detected by this method [54]. However, the technique necessitates fluorescent labeling of the EVs. Depending on the labeling method, e.g., lipophilic membrane dyes or antibodies, subpopulations of EVs may only, intentionally or unintentionally, be investigated by this technique. For high-throughput EV biomarker discovery, flow cytometry is limited but may be implemented in downstream diagnostic tests when the pathology-specific markers have already been determined (described in further detail below). More in-depth and explorative analysis of EV protein composition using proteomic techniques such as liquid chromatography fractionation in conjunction with tandem mass spectrometry (LC/MS/MS) have been reported in several studies, including studies that investigated the proteomic profile of circulating stroke EVs [55,56,57]. To date, only one study by Couch et al. investigated the proteomic profile of EVs released in the acute phase after ischemic stroke (<24 h) [56]. They found 20% of identified proteins to be significantly different in stroke EVs compared with EVs from age-matched controls. These proteins were primarily related to the acute stroke phase with elevated levels of inflammatory proteins, including C-reactive protein. Furthermore, these stroke EVs were able to activate and increase cytokine and chemokine expression in macrophages. For stroke diagnostic purposes, the identified stroke-specific EV proteins are interesting and need to be evaluated for their ability to discriminate between stroke subtypes as well as their expression consistency. Nevertheless, for the purpose of screening of many samples and potential biomarkers, methodologies that are high-throughput by design would be better-suited. One such technology, the EV Array, offers high-throughput EV capture directly from plasma, followed by multiplexed phenotyping of EV surface markers using antibodies as detection agents [58].

In recent years, several novel EV isolation and characterization techniques have been developed based on microfluidics [59,60,61]. The lab-on-a-chip nature of these microfluidic devices makes it possible to combine sequential separation, sorting and detection methods (e.g., size, immunoaffinity, acoustic force, elastic lift force) to isolate and detect EVs in small volumes of starting material—making this technology especially interesting for the possibility of EV diagnosis in an acute clinical POC setup. A microfluidic stroke diagnostic setup could be based on EV stroke biomarkers detected by the exploratory methods described above. An optimal setup should be able to isolate EVs and detect EV associated stroke markers to give a readout—preferably within minutes. Furthermore, the use of whole-blood as starting material in a POC device should reduce testing time. Wu et al. showed that by using whole-blood as the starting material, they were able to rapidly isolate EVs in a label-free manner using acoustics implemented in a microfluidic device [62], while Chen et al. produced a microfluidic device to isolate and quantify EVs from whole-blood based on filtration and magnetic bead EV enrichment [63]. The classical EV isolation and characterization techniques are well-suited for the discovery of diagnostic EVs and in-depth EV validation, however, they are time-consuming, equipment-dependent and not easily integrated in an acute POC diagnostic setup. For in-hospital diagnostics, EV immunocapture isolation by means of classical EV surface markers, followed by flow cytometry against carefully validated disease EV surface markers, offers a way to implement reproducible EV analysis of clinical samples. However, for acute POC purposes, there is a need to develop devices where EV isolation and analysis can be carried out with a small volume of whole-blood as the starting material as well as minimal handling. For this purpose, highly specialized antibodies against relevant disease-specific EV surface markers can be obtained by recombinant antibody technology [64]. Such engineered antibodies could potentially be incorporated in novel EV-capture microfluidic devices, which would make it possible to initiate biomarker measurements directly on EVs from plasma to develop a fast, within-minutes, and objective diagnostic platform. Such a device was developed by Ko et al. to measure GluR2-positive, brain-derived EVs released into the circulation after mild traumatic brain injury in a mouse model of concussion. They combined negative enrichment of background EVs on microbeads (CD45-, CD61-positive EVs) with the positive enrichment of target EVs on microbeads (CD81-positive EVs) before filtration based on size differences between background and target microbeads. They were then able to quantify GluR2-containing EVs and predict concussion with high sensitivity and specificity [65].

## 7. Extracellular Vesicles in Stroke Diagnostics

Depending on their origin, EVs have a distinct molecular profile that partly represents the phenotypic composition of the donor cell. Several cell types of the brain and circulation have been shown to release EVs into the blood during stroke. These cells include neural cells; neural progenitor cells, and blood- and vascular cells; endothelial cells, platelets, erythrocytes, granulocytes, and leukocytes including monocytes and lymphocytes [66,67,68,69,70,71,72,73,74]. Although all of these cell types have been shown to release EVs during stroke, only EVs that are released in the acute phase are of interest for acute POC diagnostic purposes. For this reason, we have only considered studies where blood samples were drawn, at the latest, 48 h after stroke onset. For all of these studies, stroke severity (NIHSS) scores were only available for Simak et al. These studies are summarized in Table 1.

In 2006, Simak and co-workers found elevated levels of a subpopulation of endothelial EVs (phosphatidylserine^+^, CD105^+^, CD41a^−^) in acute ischemic stroke compared to controls. They also reported a correlation between stroke severity and specific subpopulations of EVs with endothelial origin. The strongest correlation with ischemic lesion volume was CD54/ICAM-1 positive EVs (CD105^+^, CD54^+^, CD45^−^), while endothelial cell-derived EVs (CD105^+^, CD41a^−^, CD45^−^) correlated with long term clinical outcome. In addition, they were able to distinguish between severe and minor stroke. However, they could not differentiate patients with minor stroke from controls [73]. Similar results were reported by Chiva-Blanch et al. in a larger study, where Annexin-V-positive EVs originating from different cell types, including neural progenitor cells (CD34^+^, CD56^+^), platelets (CD61^+^), endothelial cells (CD146^+^), erythrocytes (CD235ab^+^), and leucocytes (CD45^+^), were elevated in the acute blood samples of ischemic stroke patients [67]. However, they were unable to correlate ischemic stroke etiology to the circulating EV counts in the blood samples drawn at an early timepoint. In a recent study, no general differences in EV counts between controls and stroke patients were observed, but certain subpopulations of EVs were significantly altered [78]. EVs originating from endothelial cells (CD146^+^), activated endothelial cells (CD62E^+^), activated platelets (CD62P^+^), and erythrocytes (CD235a^+^) were among the elevated EVs. Interestingly, AIS patients had a significantly higher amount of circulating EVs from activated platelets compared to patients with TIA. Using different methods (flow cytometry and ELISA), two independent studies found elevated levels of platelet-derived EVs in the acute phase of ischemic stroke [66,70]. However, none of these studies could distinguish between minor–moderate or severe stroke caused by a large vessel occlusion. In summary, these studies show promising results and increases the anticipation of the diagnostic potential of peripheral blood EVs in acute ischemic stroke.

It would be of great clinical significance to be able to differentiate ICH from LVO stroke, as their symptomatology are indistinguishable while their treatment strategies are very different. Two prior studies found elevated levels of circulating Annexin-V-positive EVs in ICH patients compared to controls [75,77]. In a more detailed study, Lackner et al. found elevated endothelial (CD105^+^, CD106^+^, CD54^+^, or CD62e^+^)-, leucocyte (CD45^+^)-, and erythrocyte (CD235^+^)-derived EVs in ICH patients [76]. These results were confirmed by Sanborn and colleagues, who also found transient elevated levels of Annexin-V-positive subpopulations of EVs from both neutrophil (CD66b^+^) and erythrocyte (CD235a^+^) origin. Furthermore, endothelial cell (CD146^+^)- and tissue factor (CD142^+^)-derived EVs were elevated during the entire 10-day study period [72]. These studies show a clear diagnostic potential of EVs, however, several of the EV populations overlap with those elevated in ischemic stroke, making those (e.g., EVs positive for CD235a^+^ erythrocytes and CD105^+^ endothelial cells) less suitable for distinguishing stroke subtypes.

Many other studies have investigated the long-term/chronic (>48 h) elevation of cell-specific EVs in stroke patients compared with controls [68,69,71,74,79,80]. This is highly relevant for understanding the role of EVs in stroke progression and pathology and how these EVs could be used in in-hospital and follow-up diagnostics. However, the reason for the elevation of EVs after days, and even months, might be completely unrelated to the acute events and could merely be indicative of a continued inflammatory response, edema, or a leaky BBB. Instead, these EV changes could be valuable as prognostic markers of long-term clinical outcome or serve as treatment-monitoring biomarkers.

## 8. EV-Derived miRNA in Stroke Diagnostics

In addition to identifying EVs based on their surface markers, EVs are packed with molecules that could function as biomarkers. In recent years, microRNAs (miRNAs) have attracted a lot of attention as they function as post-transcriptional regulators of gene expression and therefore present therapeutic potential [81]. This has also been the case for EV cargo studies, where miRNA characterization has been coupled with potential stroke treatments. Furthermore, miRNAs could function as biomarkers, as they are easily identifiable using sequencing techniques, RT-PCR or direct hybridization. With regards to POC stroke diagnostics, EV miRNA analysis of plasma obtained in the acute phase is of interest (Table 2).

Several studies have reported stroke-specific, EV-derived miRNAs. Specifically, miR-134 [82], miR-9 and miR-124 [86] were significantly increased in AIS patients in the acute phase and correlated with infarct volume and NIHSS scores. Similarly, miR-422a [84], miR-21–5p and miR-30a-5p [83] showed an initial peak expression in the acute phase followed by a downregulation in the subacute phase. These fluctuations in miRNA levels have the potential of indicating the elapsed time from stroke onset. However, variable miRNA levels introduce a risk of imperfect diagnostics depending on the time of blood sampling. In a quest to distinguish stroke types, Kalani et al. found several EV miRNAs that were capable of discriminating between AIS and ICH [88]. One of the top 20 miRNAs, miR-134 had previously been reported to be correlated to AIS, but in this study it was up-regulated in ICH patients. The study did not include a non-stroke control group, making it difficult to evaluate its potential as a stroke biomarker. That is, when selecting miRNAs for diagnostics it is important to validate their disease specificity. Van Kralingen and coworkers found that the elevation of miRNA-17-5p, miR-20b-5p and miR-93-5p (miRNA-17 family miRNAs) and miRNA-27b-3p in stroke patients compared to stroke mimic patients were linked to their underlying chronic cSVD instead of their AIS [87]. In general, these studies were conducted on fairly small patient and control groups, pointing towards the need for larger studies to evaluate and verify these findings. Most of these studies evaluate the diagnostic potential of historic stroke specific miRNAs, which predictably primarily were upregulated. Interestingly, the unbiased analysis of all EV miRNAs using NGS also shows preferential upregulation of circulating miRNAs in stroke samples. This might be due to the detection limit of NGS where lowly expressed miRNAs are not included in the analysis.

Most of the published EV miRNA studies only assess a single or a few EV-derived miRNAs for stroke identification. However, evaluating several EV miRNAs, as exemplified by Kalani and colleagues [88], could increase the specificity and sensitivity of stroke diagnosis and could help to differentiate between stroke types. Microfluidic chips are being developed to unleash the potential of miRNA evaluation in POC testing even in multiplex formats that allows simultaneous miRNA estimation [89]. This chip has a detection limit of femto- to picomolar, while the assay time is about 20 min, which in some cases would be too long for stroke diagnosis. In addition, EV isolation and miRNA purification will further prolong the assay time, showing the need for additional development in microfluidic chip design.

## 9. Conclusions

Minimizing treatment delays in stroke patients is of utmost importance, as treatment efficacy is highly time-dependent. As current diagnosis depends on neuroimaging, circulating molecular biomarkers are central in the hunt for fast and reliable POC diagnosis of stroke types. Thus, blood-derived EV stroke biomarkers have great potential in acute POC diagnostic tests. EVs present an interesting and, to date, unexploited resource for blood-based diagnostics. Current developments in utilizing EVs for diagnostics in, e.g., cancer, can now be utilized as diagnostic aids in other disease states. However, with time as the limiting factor in stroke, novel technological developments are needed before EV-based diagnostics can be implemented in the prehospital phase. The development of reliable POC stroke diagnostics in the acute setting will have a huge impact on prehospital delay, as each patient can be directed to the nearest hospital with the optimal treatment capabilities, and might even enable the initiation of neuroprotective treatment in the ambulance. Ultimately, this could lead to improved functional outcome for stroke patients.

## Figures and Tables

**Table 1:** Acute EV Responses to Stroke.

Disease	Results	Origin of EVs	Analysis Method	Patients/Controls	Time Since Onset	Ref.
AIS—minor (NIHSS < 5) and moderate-severe (NIHSS ≥ 5)	Endothelial cell EVs are linked to severity, lesion volume and outcome of AIS.	Endothelial cell	Flow cytometry	20 minor stroke 21 moderate–severe stroke/23 age-matched controls	37 h on avg.	Simak et al. [73]
AIS	AIS increases EV shedding from blood and vascular compartment cell and neural precursor cell	Endothelial cells, Platelets, erythrocytes, Leukocytes, monocytes lymphocytes and Neural precursor cells	Flow cytometry	44 AIS/44 controls (age-matched, high cardiovascular risk subjects—no documented vascular disease)	Max 48 h	Chiva-Blanch et al. [67]
AIS (LAA and cSVD)	Platelet EVs are elevated in all groups, compared with control	Platelets	Flow cytometry	112 AIS incl. LAA and cSVD stroke/35 controls	Max 48 h	Chen et al. [66]
AIS (LVO and cSVD)	Platelet EVs are significantly elevated in LVO and cSVD	Platelets	ELISA	34 cSVD stroke, 41 LVO/61 patients with no apparent cerebral vascular lesions	Max 24 h	Kuriyama et al. [70]
ICH	The EVs show a distinct temporal profiling depending on their origin.	Endothelial cells, erythrocytes, neutrophils	Flow cytometry	22 ICH/13 controls	Max 48 h	Sanborn et al. [72]
ICH	Annexin V positive EVs are elevated in ICH compared to controls at admission	Undetermined	Pro-thrombinase assay	38 ICH/10 controls	Max 8.5 h	Huang et al. [75]
ICH	Increase in endothelial, leucocyte and erythrocyte EVs (not platelet)	Endothelial cells, leucocytes erythrocytes	Flow cytometry	20 ICH/22 controls	Max 48 h	Lackner et al. [76]
ICH	Annexin V positive EVs are elevated in ICH compared to controls at admission	Undetermined	Pro-thrombinase assay	86 ICH/30 controls	Max 6 h	Dong et al. [77]

Abbreviation: AIS: Acute ischemic stroke, ICH: Intracerebral hemorrhage, EVs: extracellular vesicles, LVO: Large vessel occlusion, LAA: large artery atherosclerosis, cSVD stroke: cerebral small vessel disease stroke, avg.: average.

**Table 2:** Acute Responses to Stroke of miRNAs.

Stroke Type	miRNA	Expression in Stroke	Source	Analysis	Patients/Controls	Time from Onset	Ref.
AIS (NIHSS: 8)	miR-134	Upregulated	Serum	ExoQuickexosome isolation, qRT-PCR	50 AIS/50 controls	Max 24 h	Zhou et al. [82]
AIS (NIHSS: 6)	miR-21-5 p and 30a-5p	Upregulated in hyper acute phase	Plasma	QIAGEN exoRNeasy, NTA, Flow cytometry, qRT-PCR	143 AIS/24 non-stroke controls	Max 6 h	Wang et al. [83]
AIS (NIHSS: N/A)	miR-422a	Upregulated	Plasma	qRT-PCR	55 AIS/25 age- and sex-matched controls	1–3 days	Li et al. [84]
AIS (NIHSS: 3)	miR-223	Upregulated	Blood	ExoQuickexosome isolation, qRT-PCR	50 AIS/33 age- and sex-matched controls	72 h	Chen et al. [85]
AIS (NIHSS: 8)	miR-9 and miR-124	Upregulated	Serum	ExoQuickexosome isolation qRT-PCR	65 AIS/66 non-stroke controls	16.5 h on avg.	Ji et al. [86]
AIS&cSVD (NIHSS: 4)	miRNA-17 Family and miR-27b-3p	Upregulated (linked to chronic cSVD)	Serum	Thermo Fisher exosome isolation reagent, qRT-PCR	139 AIS and chronic cSVD/39 non-stroke (cSVD) patients	48 h post AIS	Van Kralingen et al. [87]
ICH, AIS, SAH (NIHSS: N/A)	miR-27b-3p and miR-146b-5p, i.a.	Upregulated	Plasma	QIAGEN exoRNeasy, NGS	21 AIS, 17 SAH, 19 ICH	Max 24 h	Kalani et al. [88]

Abbreviation: AIS: Acute ischemic stroke, ICH: Intracerebral hemorrhage, miRNA: microRNA, cSVD: cerebral small vessel disease, NIHSS: national institute of health stroke scale (mean), NGS: next generation sequencing, i.a.: among others, SAH: Subarachnoid hemorrhage, avg.: average.
